# Determination of the content of rosmarinic acid by HPLC and analytical comparison of volatile constituents by GC-MS in different parts of *Perilla frutescens* (L.) Britt

**DOI:** 10.1186/1752-153X-7-61

**Published:** 2013-04-01

**Authors:** Jing Liu, Yuklam Wan, Zhongzhen Zhao, Hubiao Chen

**Affiliations:** 1 School of Chinese Medicine, Hong Kong Baptist University, Hong Kong Special Administrative Region, Hong Kong, People’s Republic China

**Keywords:** *Perilla frutescens*, Quantitative analysis, Rosmarinic acid, Qualitative analysis, Volatile constituents

## Abstract

**Background:**

*Perilla frutescens* (L.) Britt. is not only an edible plant but also a traditional medicinal plant commonly used for treating common cold. It is widely cultivated in southern China. The anatomical parts of *P. frutescens* that are recorded as medicines in the Chinese material medica are: Perillae Caulis, Perillae Folium and Perillae Fructus, which are the dried stems, the dried leaves and the dried ripe fruits, respectively. Rosmarinic acid is one of major polyphenolic ingredients found in all three Perillae Caulis, Perillae Folium and Perillae Fructus. The characteristic volatile oil of *P. frutescens* is believed to be another essential composition of the herb, giving its wide range of use.

**Results:**

A simple, rapid and accurate HPLC-DAD method was set up, suitable for the assay of rosmarinic acid in Perillae Fructus, Perillae Folium and Perillae Caulis. 12 batches of Perillae Caulis, 12 batches of Perillae Folium and 13 batches of Perillae Fructus from 8 different regions of mainland China and Hong Kong herbal markets were collected for evaluating the quality of *P. frutescens*. Results showed that Perillae Folium typically had the highest content of rosmarinic acid. Certain macroscopic characteristics were related to the concentration of rosmarinic acid. The volatile components were identified and compared in Perillae Fructus, Perillae Folium and Perillae Caulis by gas chromatography–mass spectrometry (GC-MS). Extracts were prepared by steam distillation. Twelve, seventeen and nine compounds were identified and accounted for 69.71%, 50.54% and 81.73% of all identified peak areas in Perillae Caulis, Perillae Folium and Perillae Fructus respectively. The identified components were analyzed for comparison of Perillae Caulis, Perillae Folium and Perillae Fructus more effectively.

**Conclusions:**

This work provides a means by which samples of various parts of *P. frutescens* can be evaluated in terms of their pharmacologically active components. It should be of value in the efficient exploitation of *P. frutescens* plant material in clinical applications and drug development.

## Background

*Perilla frutescens* (L.) Britt. (Lamiaceae) is an edible plant frequently used as a fresh vegetable, to process pickles, and as one of the most popular garnishes and food colorants in Asian countries, particularly China, Korea and Japan. *P. frutescens* is also a commonly used herbal medicine in China, where it is known as “Zisu”. *P. frutescens* has been widely cultivated in southern China for centuries. In the Chinese Pharmacopeia 2010 [1], the dried stem of *P. frutescens* is recorded as Perillae Caulis (PCa), the dried leaf of *P. frutescens* is recorded as Perillae Folium (PFo) and the dried seed of *P. frutescens* is recorded as Perillae Fructus (PFr).

**Figure 1:**
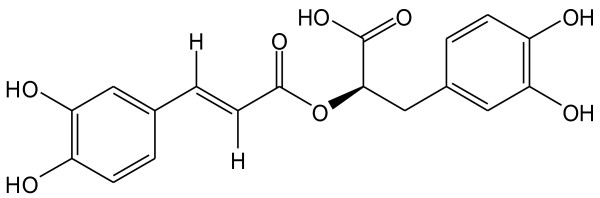
Molecular structure of rosmarinic acid.

Modern laboratory studies confirm the pharmacological effects of *P. frutescens*. The stems of *P. frutescens* are reported to have the effects on the contraction of colon smooth muscle cells of rats with lower limb ischemic reperfusion [2]. The leaves of *P. frutescens* are proved to be detoxicant, antitussive, antipyretic and antibiotic [3,4]; they are usually used as a folk medicine for treating intestinal disorders and allergies in traditional Chinese medical practice [5]. The seeds of *P. frutescens* are shown to have antimicrobial and inhibitory activities against *α*-glucosidase and aldose reductase [6,7]. Rosmarinic acid (RA, Figure 1) has been proven to be the main biological polyphenolic compound found in the stems, leaves and seeds of *P. frutescens*[8-10]. Determination of RA and other phenolic compounds in *P. frutescens* for quality control is documented [11-13], however, to our knowledge, no studies have been published on the quantitative analysis and comparison of RA in all three parts of *P. frutescens*. Hence, it is necessary to establish some simple, economical and accurate methods for the quality assessment of RA in the stems, leaves and seeds of *P. frutescens*.

The volatile oil of *P. frutescens* is believed to be another essential part of the herb. It was claimed to have anti-inflammatory, anti-aging, anti-hyperlipidemia and antimicrobial activities [14-18]. Apart from pharmaceutical and eatable use, it was also applied to produce perfume, soap, detergents and cosmetics. In the present work, gas chromatography–mass spectrometry (GC-MS), heuristic evolving latent project ions method (HELP) and chemometric resolution methods (CRMs) have been used for qualitative and quantitative analyses of *P. frutescens*[19-22]. So far, no systematic comparative study has been done on the volatile oils of different parts of *P. frutescens*. Such research cannot only be helpful for finding out the possibly common and different chemical constituents but also provide the scientific evidence with current practical use of volatile oil in *P. frutescens*.

Our work aimed at investigating of contents of RA and volatile compositions which are known to be the most important natural active ingredients in different medicinal parts (stems, leaves and seeds) of *P. frutescens*. We carried out quantitative and qualitative analysis of the samples collected from various herbal markets by HPLC and GC-MS respectively. A further knowledge of clinical medication of *P. frutescens* can be obtained by comparing the differences and similarities among the analysis samples.

### Experimental

#### Plant materials

The stems, leaves and seeds of *P. frutescens* were bought from herb markets in mainland China and Hong Kong. All of them were collected from major cultivation regions. Table 1 was shown the details including collected market location, received date and morphological descriptions for each sample. The plant materials were identified by Dr. CHEN Hu-Biao, School of Chinese Medicine, Hong Kong Baptist University. The voucher specimens are deposited at the Herbarium, School of Chinese Medicine, Hong Kong Baptist University.

**Table 1:** The morphological descriptions and assay results of plant materials

**Crude drug**	**Code No.**	**Locality**	**Date received**	**Concentration of Rosmarinic acid (mg/kg) (n = 2)**	**Morphological description**		
					**Cutting**	**Color of surface**	**Color of cross-section**
**Perillae Caulis**	PCa-01	Beijing	2011.07.15	343.55	straight	purplish-brown, green	cream-white
	PCa-02	Sichuan	2011.08.16	1918.35	straight	purple, green	cream-white
	PCa-03	Guangxi (cultivated)	2011.08.17	3081.25	oblique	purplish-brown	cream-yellow
	PCa-04	Guangxi (cultivated)	2011.08.17	1318.00	oblique	dark brown	pale yellow
	PCa-05	Hunan	2011.08.22	1180.35	straight	purplish-brown	cream-white
	PCa-06	Guangdong	2011.08.23	2183.50	oblique	purplish-brown	cream-yellow
	PCa-07	Jiangsu	2011.09.09	247.95	straight	grayish-green	cream-white
	PCa-08	Hong Kong	2011.09.09	2611.65	oblique	purplish-brown, green	cream-yellow
	PCa-09	Hong Kong	2011.10.26	1445.60	oblique	dark brown	cream-yellow
	PCa-10	Hong Kong	2011.10.26	1221.65	oblique	dark brown	pale yellow
	PCa-11	Hong Kong	2011.10.26	1457.85	oblique	purplish-brown	pale yellow
	PCa-12	Hebei	2011.11.03	not detected	straight	grayish-green	cream-white
**Crude drug**	**Code No.**	**Locality**	**Date received**	**Concentration of Rosmarinic acid (mg/kg) (n = 2)**	**Morphological description**		
					**Shape of leaves**	**Color of surface**	**Odor**
**Perillae Folium**	PFo-01	Beijing	2011.07.15	934.95	small, fragmented, rolled-up	green	none
	PFo-02	Sichuan	2011.08.16	2371.25	small, fragmented, rolled-up	purplish green	none
	PFo-03	Guangxi (wild)	2011.08.17	8008.70	small, fragmented, rolled-up	green	none
	PFo-04	Guangxi (cultivated)	2011.08.17	2679.25	small, fragmented, rolled-up	purplish green	none
	PFo-05	Hunan	2011.08.22	4286.60	medium, fragmented, rolled-up	brownish green, grayish green and little purple	none
	PFo-06	Guangdong	2011.08.23	12394.00	large, whole, flat	brown, purple and green	sweet
	PFo-07	Jiangsu	2011.09.09	833.10	small, fragmented, rolled-up	green	none
	PFo-08	Hong Kong	2011.09.09	7415.85	large, whole, flat	purple and green	sweet
	PFo-09	Hong Kong	2011.10.26	6612.95	large, whole, flat	purple and green	sweet
	PFo-10	Hong Kong	2011.10.26	14978.65	large, whole, flat	purple and green	sweet
	PFo-11	Hong Kong	2011.10.26	5700.60	large, whole, flat	purple and green	sweet
	PFo-12	Hebei	2011.11.03	1558.45	small, fragmented, rolled-up	dark green with little brown	none
**Crude drug**	**Code No.**	**Locality**	**Date received**	**Concentration of Rosmarinic acid (mg/kg) (n = 2)**	**Morphological description**		
					**Color**	**Diameter (mm)**	
**Perillae Fructus**	PFr-01	Beijing	2011.07.15	2109.75	dark brown	1.5-2	
	PFr-02	Sichuan	2011.08.16	1167.55	brown and grayish white	<1.5	
	PFr-03	Guangxi (cultivated)	2011.08.17	3308.35	dark brown	1.5-2	
	PFr-04	Guangxi (cultivated)	2011.08.17	3471.30	brown	1.5-2	
	PFr-05	Guangxi	2011.08.17	1347.45	pale brown	1.5-2	
	PFr-06	Hunan	2011.08.22	1033.00	dark brown	<1.5	
	PFr-07	Guangdong	2011.08.23	2110.95	brown	1.5-2	
	PFr-08	Jiangsu	2011.09.09	2007.20	grey and brown	<1.5	
	PFr-09	Hong Kong	2011.10.26	1700.85	dark brown and grey	<1.5	
	PFr-10	Hong Kong	2011.10.26	1832.70	dark brown and grey	<1.5	
	PFr-11	Hong Kong	2011.10.26	1949.70	brown	<1.5	
	PFr-12	Hong Kong	2011.10.26	2247.60	brown	<1.5	
	PFr-13	Hebei	2011.11.03	2223.95	grey and brown	1.5-2	

#### Reagents and apparatus

HPLC-grade methanol, *n*-hexane, acetonitrile, ethanol, sodium sulphate were purchased from RCI Lab-scan (Bangkok, Thailand). There were also trifluoroacetic acid (TFA, International Laboratory, USA). Water was purified using a Milli-Q water system (Millipore; Bedford, MA, USA). Rosmarinic acid (>99% purity) was bought from Chengdu Biopurify Phytochemicals Ltd. (Chengdu, China).

Electronic balance (Adventurer^®^), Centrifuge 5810 (Eppendorf R-114), Crest ultrasound meter, Heater (Electromantle 10575592), quick-fit apparatus, Agilent 1100 series HPLC system equipped with a quaternary gradient pump unit, a DAD detector, and an autosampler (0.1-100 μL). The analytical column used was a Grace Alltima-C_18_ column (250 mm × 4.6 mm, 5 μm, Phenomenex, USA). A Shimadzu QP2010 GC-MS (Shimadzu, Japan) equipped with an AOC-20i autosampler was used. A DB-5 ms column (0.25 μm × 30.0 m × 0.25 μm) was used in the volatile oil analysis.

### Quantitative analysis of rosmarinic acid by HPLC-DAD

#### Sample preparation

The dried samples were powdered by a mill and screened through a 380 μm sieves. Each sample of fine powder (0.5 g) was accurately weighed and extracted twice by 10 mL methanol-H_2_O (7:3) by ultrasonication at room temperature for 30 min. After centrifugation, the combined solution was transferred into a 25-mL volumetric flask and made up to volume with 70% methanol and filtered through a syringe filter (0.2 μm, Alltech, Beerfield, IL, USA). An aliquot of 10 μL of the filtrate was injected into HPLC for analysis.

#### HPLC conditions

The analysis of RA was carried out by HPLC. 330 nm was selected as the wavelength for UV detection. Elution was carried out at a flow rate of 1.0 mL/min at 25°C. Two mobile phases, A and B were used. Mobile phase A was 0.1% (v/v) formic acid solution in water, while mobile phase B was acetonitrile. A ratio of 88% A and 12% B was applied in the first 30 min. After 30 min, a ratio of 80% A and 20% B was used for the next 15 min. Finally, 70% A and 30% B were used after 45 min for an additional 15 min.

### Qualitative analysis of volatile components by GC-MS

#### Extraction of volatile components

The volatile components from Perillae Caulis (PCa-08), Perillae Folium (PFo-08) and Perillae Fructus (PFr-09) collected from the same market in Hong Kong were isolated by steam distillation according to the standard extraction method for the determination of volatile oils as stated in Chinese Pharmacopoeia 2010 [1]. 40 g of ground samples were extracted by 300 mL of distilled water under reflux for 6 h. The obtained volatile oil was recovered with *n*-hexane, dried over anhydrous sodium sulphate and finally stored in dark glass bottle at 4°C prior to GC-MS analysis.

#### GC-MS conditions

Initial temperature was 60°C and maintained for 1 min. Temperature rose to 200°C at a rate of 4°C/min and held for 2 min. Then, the temperature reached 260°C and was held for 3 min. The rate was 10°C/min. Helium was the carrier gas. Pressure was 57.4 kPa. Total flow and column flow were 50.0 mL/min and 1.0 mL/min respectively. Linear velocity was 36.5 cm/sec. Purge flow was 3.0 mL/min. Total program time was 45 min. Injection volume was 1 μL. Injector, interface and ion-source were kept at 250°C, 250°C and 200°C, respectively. Solvent cut time was 5 min. 1250 scan speed was applied from 5 min to 45 min. Through comparing the TIC with NIST mass spectral database, probable compound for each peaks were identified. Only the compounds with compatibility greater than 90% were recorded.

## Results and discussion

### Optimization of quantitative analysis of rosmarinic acid

The extraction solvents including methanol, 70% methanol, ethanol and 70% ethanol were optimized. (Additional file 1: Table S1) illustrated that ethanol could not extract RA from PCa-01, PFo-01 or PFr-01. 70% methanol extracted the highest amount of RA from the three parts of *P. frutescens* as the peak area-to-weight ratio was the highest as compared to using 70% ethanol and methanol as extraction solvents. Even though extraction by 70% ethanol showed a higher peak area-to-weight ratio than extraction by 70% methanol in PCa the difference was not significant. Therefore, 70% methanol was adopted as the best extraction solvent.

The extraction methods including sonication and reflux were compared in order to establish and standardize an effective method for the assay. Selected samples PCa-01, PFo-01 and PFr-01 were extracted separately by sonication for 30 min, reflux for 1 h and for 2 h using 70% methanol as extraction solvent. The results of peak area of RA based on HPLC analysis as shown in (Additional file 2: Table S2) indicated that reflux extraction had slightly higher extraction efficiency than sonication. Due to the simple procedure and similar extraction efficiency of RA, sonication for 30 min was eventually chosen as the best extraction method.

Times of extraction were also investigated. By comparing the peak area of RA at different times of extraction as shown in (Additional file 3: Table S3), no RA was detected in the third extraction. Extraction twice is good enough for analysis.

**Figure 2:**
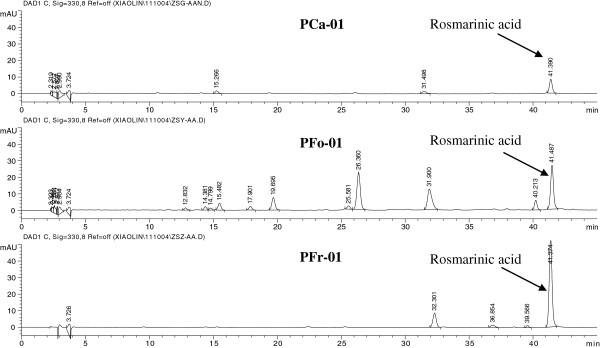
HPLC chromatograms of Perillae Caulis (PCa-01), Perillae Folium (PFo-01) and Perillae Fructus (PFr-01).

HPLC condition was optimized based on the optimized extraction method. 20°C, 25°C and 30°C of the HPLC column temperature were selected for the investigation. Referring to the chromatograms, the symmetry of RA peak was most nearest to 1 under 25°C in all three PCa-01, PFo-01 and PFr-01 samples. Several HPLC gradient programs were designed for the optimization. The selected program described in HPLC Equipment and Conditions part was shown to have the best symmetry of RA peak. Figure 2 shows typical HPLC chromatograms of PCa-01, PFo-01 and PFr-01.

### Calibration curve, reliabilities and recoveries of HPLC method

A series of standard solutions ranging from 1.045 to 418.000 mg/L in concentration were tested to determine the calibration curve. The regression equation for RA was calculated in the form of *y* = *ax* + *b*, where *y* and *x* were peak area and amount of compound injected, respectively. The limit of detection (LOD) and limit of quantification (LOQ) were determined at a signal-to-noise (S/N) ratio of 3 and 10, respectively. The results are shown in Table 2.

**Table 2:** Results of regression analysis on calibration and detection limits

**Marker**	**Concentration (mg/L)**	**Equation**	**Correlation coefficient (r**^**2**^**)**	**LOD (mg/ L)**	**LOQ (mg/ L)**
Rosmarinic acid	1.045, 2.090, 5.225, 20.900, 209.000, 418.000	y = 59.037x - 0.5153	0.9999	0.0628	0.3120

Five replicates of each of PCa-01, PFo-01 and PFr-01 samples were analyzed respectively for repeatability study. Results showed that the relative standard deviation (RSD) values of RA in PCa-01, PFo-01 and PFr-01 were 2.98%, 2.27% and 4.71%, respectively, confirming that the method was appropriate for analysis. The RSD of 5 replicate injections of standard RA solutions (20.9 mg/L) was also investigated. The RSD value was 0.71%, representing the good precision.

The recovery test was used to evaluate the accuracy of this method. Accurate amounts of RA were added to 0.5 g of samples of PCa-01, PFo-01 and PFr-01 in triplicate; then the spiked samples were extracted and analyzed as described in the sample preparation section. The results are shown in Table 3. The percent of average recoveries ranged from 91.12 to 100.46%. The RSD values for the recovery test were within the range 0.37-1.88%.

**Table 3:** **Recoveries of the described method for RA in different parts of *P. frutescens***

**Sample**	**Trial**	**Sample weigh (g)**	**Spiked amount (mg)**	**Cal. conc (mg/L)**	**Cal. total amount (mg)**	**Amount in sample (mg)**	**Cal. spiked amount (mg)**	**Recovery (%)**
**Perillae Caulis (PCa-01)**	1	0.5038	0.1672	12.7781	0.3195	0.1660	0.1535	91.79
	2	0.5019	0.1672	12.7081	0.3177	0.1653	0.1524	91.12
	3	0.5014	0.1672	12.7228	0.3181	0.1652	0.1529	91.44
	**Mean**	91.45						
	**SD**	0.34						
	**RSD (%)**	0.37						
**Perillae Folium (PFo-01)**	1	0.5023	0.4703	37.0332	0.9258	0.4600	0.4658	99.05
	2	0.5026	0.4703	37.2100	0.9302	0.4603	0.4700	99.93
	3	0.5027	0.4703	37.3131	0.9328	0.4604	0.4725	100.46
	**Mean**	99.81						
	**SD**	0.71						
	**RSD (%)**	0.71						
**Perillae Fructus (PFr-01)**	1	0.5043	1.0090	83.4811	2.0870	1.1388	0.9482	93.98
	2	0.5021	1.0090	83.7794	2.0945	1.1338	0.9607	95.21
	3	0.5043	1.0090	84.9101	2.1228	1.1388	0.9840	97.52
	**Mean**	95.57						
	**SD**	1.80						
	**RSD (%)**	1.88						

The above assay results indicate that this HPLC-DAD method is accurate, reproducible, precise and sensitive enough for quantitative evaluation of RA in different parts of *P. frutescens*. As stated in Chinese Pharmacopeia 2010 [1], the methods for quantitative evaluation of RA in PCa and PFr are similar, however, the extraction solvent, extraction method and the HPLC condition used in these two methods are different. It is a huge convenience that our developed method is suitable for quantitative evaluation of RA in all PCa, PFr and PFo.

### Comparison of content in different parts of *P. frutescens*

The newly established HPLC-DAD assay method was used to assess the amount of RA in different samples of the same botanical part. The quantitative analytical results are summarized in Table 1. Generally, the concentration of RA in PFo was more than twice as much as that in either PFr or PCa. Samples of PFo had an average RA content of 5647.86 mg/kg while the average concentrations of RA in PFr and PCa were 2039.26 mg/kg and 1417.48 mg/kg respectively. The amount of RA in PFr was more than that in PCa which was a normal expectation. Referring to Chinese Pharmacopeia 2010 [1], the content of RA in PFr should not lower than 0.25% and that in PCa should not below 0.1%. The result showed that concentration of RA in PFo was 2.7 times more than that in PFr and 3.5 times more than that in PCa.

### Relationship between content of rosmarinic acid and macroscopic characteristics

Quality assessment based on macroscopic characteristics is widely used in the herbal markets in China [23]. In this study, we tried to investigate the relationship between macroscopic characteristics and RA content and to establish quality control for the three medicinal parts of *P. frutescens*.

Sample PCa-03 collected from Guangxi had the highest concentration of RA (3081.25 mg/kg), while sample PCa-07 collected from Jiangsu had the lowest (247.95 mg/kg). From our observation, the appearance and color of the crude herbs directly correlated with RA concentration. For PCa, of which samples were cut into oblique or straight slices, slices that were rich cream-yellow had more RA than pale-yellow slices. The surface color of PCa also reflected its RA concentration. From our results, PCa with purple and brown surfaces had the highest concentration, while dark brown samples had somewhat less and grayish green samples had the lowest concentration.

For PFo, the concentrations of RA in the twelve batches ranged from 833.10 mg/kg to 14978.65 mg/kg. The great amount of RA was found in sample PFo-10 collected from Hong Kong. The least amount was found in sample PFo-07 collected from Jiangsu. In these samples, leaf color correlated with amount of RA. Leaf color varied greatly in the 12 samples. To help organize our observations, we classified this color variation into four categories, namely, green, purplish-green, purple with green, brown with green. On average, purple with green leaves had the most RA. The odor of leaves was also investigated. Samples with stronger smell had a significantly higher amount of RA than the samples without odor. We examined shapes of leaves to see if there was any correlation between shape and RA content. Our samples of PFo were of two distinct forms: leaves were either rolled up, so that they easily crumbled into small fragments; or the leaves were flat and whole. In our investigation, samples which comprised whole leaves consistently had significantly more RA than the rolled-up samples had.

Sample PFr-04 collected from Guangxi had the highest concentration of RA (3471.30 mg/kg) while sample PFr-06 had the lowest (1033.00 mg/kg). From the assay results, we could observe that brown fruits usually had higher RA concentration; larger samples (> 1.5-2 mm diameter) had more RA than smaller samples (< 1.5 mm diameter).

To summarize, PCa with high RA concentration commonly had the following characteristics: cream-yellow cross-section surface, large stem diameter and surface color of purple and brown. These characteristics matched the regulations of Chinese Pharmacopeia for this herbal medicine. For PFo, samples with large whole leaves, purple-and-green, sweet smelling leaves had more RA. PFr with brown color and large diameter contained more RA.

### Comparison of mainland China and Hong Kong samples

As the crude drugs were collected from different herbal markets both in mainland China and Hong Kong showed variation, the quality of different parts of *P. frutescens* from the two regions was compared.

The concentration of RA in PCa collected from mainland China markets fluctuated while that from Hong Kong markets was relatively stable. PCa collected from mainland China markets had a larger range of RA (247.95 mg/kg-3081.25 mg/kg) compared to PCa collected in Hong Kong markets (1221.65 mg/kg-2611.65 mg/kg). It indicated that quality of PCa in mainland China markets was not good as that of Perillae Caulis in Hong Kong markets.

Moreover, we noticed that samples of PFo collected from Hong Kong were all large whole leaves and they were sold in a bundle. Besides, they had sweet odor. Their surface color was also purple and green. In comparison, samples of PFo collected from mainland China were rolled leaves, without odor and the surface color was mainly dark green and brown. Results shown in Table 1 clearly indicated that the quality of PFo collected from Hong Kong was higher than that of PFo collected from mainland China in terms of RA concentration. These results are similar to those for PCa. That is, the concentration of RA in PFo collected from mainland China fluctuated greatly whereas that in PFo collected from Hong Kong was relatively steady.

With regard to PFr, the size of PFr collected in Hong Kong was smaller than that collected in mainland China; however, the contents of RA in PFr collected from Hong Kong markets were more consistent as compared to those in PFr collected from mainland China.

Generally speaking, the qualities of all of the crude drugs derived from *P. frutescens*, namely PCa, PFo and PFr collected in Hong Kong markets were better than those collected in mainland China markets based on the concentration of RA. Also, the contents of RA in crude drugs collected from Hong Kong markets were more consistent. In contrast, the contents of RA in samples from mainland China markets fluctuate greatly. It may indicate that the Chinese Herbal Medicine markets in Hong Kong market had better sources of PCa, PFo and PFr and had a standardized quality control.

### Optimization of qualitative analysis of volatile components

**Figure 3:**
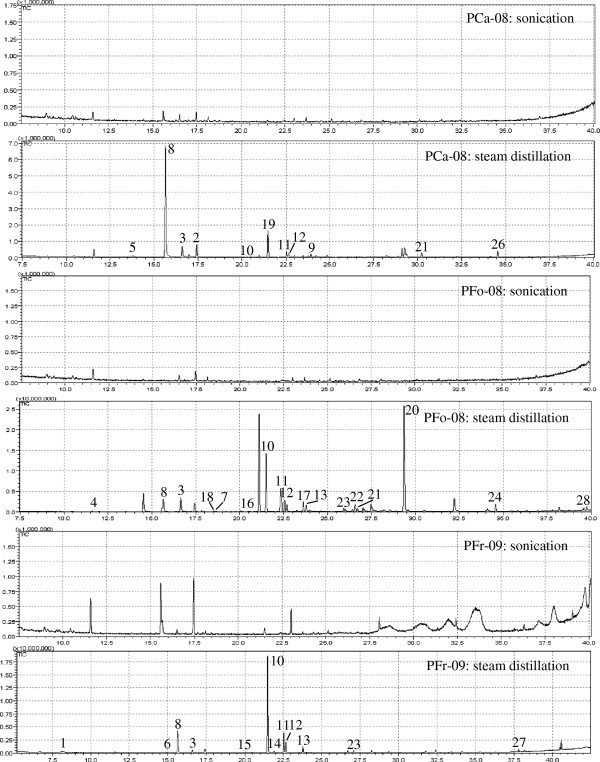
**GC-MS chromatograms of volatile components extracted from different parts of *P. frutescens *by different extraction methods.**

Steam distillation is the commonly used method to extract the volatile oil from PCa, PFo and PFr [20-22]. It is easy, efficient and relatively inexpensive. Sonication, supercritical fluid extraction (SFE), simultaneous distillation and solvent extraction (SDE), microwave-assisted steam extraction, and solid-phase microextraction (SPME) are also used to extract volatile oil from *P. frutescens*[19,24-26]. Sonication has various advantages such as high extraction efficiency, short extraction time, low extraction temperature, low cost and easy control. In our investigation, efficiency of sonication was chosen to compare with that of steam distillation. For sonication extraction, 40 mL of *n*-hexane was used as extraction solvent and the mixture was under sonication for 30 min. As shown in Figure 3, more peaks were found in PCa, PFo and PFr via steam distillation than via sonication. Steam distillation was eventually selected as the extraction method. The volatile oils extracted from PFr, PFo and PCa were diluted to an appropriate concentration so as to obtain a better chromatogram before GC-MS analysis,

### Comparison of volatile components in different parts of *P. frutescens*

By steam distillation, 0.15 mL, 0.40 mL and 0.20 mL of volatile oils were extracted from 40 g PCa-08, PFo-08 and PFr-09, respectively. PFo has the greatest amount of volatile oil. Only the compounds with compatibility greater than 90% were identified. By comparing the TIC chromatograms of PFr, PFo and PCa with NIST mass spectral database as well as spectral data and retention indices from the literature [12,20,21,27,28], probable compounds of certain peaks were identified and listed in Table 4. 2-hexanoylfuran (8), asarone (20) and *β*-caryophyllene (10) were identified as the most abundant of the volatile components and accounted for 43.54%, 23.91% and 45.47% of all identified peak area in PCa, PFo and PFr, respectively. The resolved mass spectra of these three compounds were shown in (Additional file 4: Figure S1). The volatile components common to PCa, PFo and PFr were the five chemicals: 1-cyclohexane-1-carboxaldehyde (3), 2-hexanoylfuran (8), *β*-caryophyllene (10), *β*-farnesene (11) and 1,4,7-cycloundecatriene-1,5,9,9-tetramethyl-zzz (12). The compounds *α*-farnesene (13) and caryophyllene oxide (22) were found in PFr and PFo but not found in PCa.

**Table 4:** Volatile components identified in Perillae Caulis, Perillae Folium and Perillae Fructus

**Number**	**Compound**	**Relative molecular weight**	**Relative content (%)**		
			**Perillae Caulis**	**Perillae Folium**	**Perillae Fructus**
1	*β*-cymene	134			0.54
2	anisole	148	1.53		
3	1-cyclohexane-1-carboxaldehyde	150	4.77	3.38	1.76
4	*β*-linalool	154		0.15	
5	*α*-terpineol	154	0.73		
6	methyl thymyl ether	164			0.49
7	2-methoxy-3-propenyl-phenol	164		0.16	
8	2-hexanoylfuran	166	43.54	2.49	12.25
9	*α*-curcumene	202	0.79		
10	*β*-caryophyllene	204	0.84	10.15	45.47
11	*β*-farnesene	204	2.04	2.00	9.37
12	1,4,7-cycloundecatriene-1,5,9,9-tetramethyl-zzz	204	1.63	1.29	5.20
13	*α*-farnesene	204		1.61	3.02
14	*α*-bergamotene	204			0.71
15	*α*-copaene	204			0.55
16	*β*-elemene	204		0.25	
17	1,6-cyclodecadiene	204		0.19	
18	elixene	204		0.13	
19	caryophyllene	204	9.54		
20	asarone	208		23.91	
21	curlone	218	1.80		
22	caryophyllene oxide	220		0.73	1.41
23	spathulenol	220		1.59	
24	trans-nerolidol	222		0.48	
25	1,2-benzenedicarboxylic acid	278		1.46	
26	phthalic acid	278	2.50		
27	hexadecanoic acid	284			0.96
28	phytol	296		0.57	
**Total percentage of all identified peaks area**			69.71	50.54	81.73

The main volatile compounds found in PCa were 2-hexanoylfuran (43.54%), caryophyllene (9.54%) and 1-cyclohexene-1-carboxaldehyde (4.77%). Eleven peaks were found due to its compatibility greater than 90%. Eight of them had been reported previously, they were 1-cyclohexene-1-carboxaldehyde (3), *α*-terpineol (5), 2-hexanoylfuran (8), *α*-curcumene (9), *β*-caryophyllene (10), *β*-farnesene (11), caryophyllene (19) and curlone (21). Meanwhile, three compounds were found for the first time in *P. frutescens* namely, anisole (2), 1,4,7-cycloundecatriene-1,5,9,9-tetramethyl-zzz (12) and phthalic acid (26).

In PFo, asarone (23.91%), *β*-caryophyllene (10.15%) and 1-cyclohexane-1-carboxaldehyde (3.38%) were the major volatile components found. We identified seventeen peaks successfully which accounted for 50.54% of all peak area. Eleven of these compounds had been reported previously; they are: 1-cyclohexane-1-carboxaldehyde (3), *β*-linalool (4), 2-hexanoylfuran (8), *β*-caryophyllene (10), *α*-farnesene (13), *β*-elemene (16), germacrene (17), asarone (20), caryophyllene oxide (22), trans-nerolidol (24) and phytol (28). Six compounds were firstly reported in *P. frutescens* by our findings, they are 2-methoxy-3-propenyl-phenol (7), *β*-farnesene (11), 1,4,7-cycloundecatriene-4,5,9,9-tetramethyl-zzz (12), elixene (18), spathulenol (23) and 1,2-benzenedicarboxylic acid (25).

The main volatile components found in PFr were *β*-caryophyllene (45.47%), 2-hexanoylfuran (12.25%) and *β*-farnesene (9.37%). Twelve peaks, which accounted for 81.73% of the all peak area, were identified. Nine of them had been reported previously; they are: *β*-cymene (1), 1-cyclohexane-1-carboxaldehyde (3), 2-hexanoylfuran (8), *β*-caryophyllene (10), *α*-farnesene (13), *α*-bergamotene (14), *α*-copaene (15), caryophyllene oxide (22) and hexadecanoic acid (27). Three are reported here in *P. frutescens* for the first time; they are: methyl thymyl ether (6), 1,4,7-cycloundecatriene-1,5,9,9-tetramethyl-zzz (12) and *β*-farnesene (11).

## Conclusions

In this work, a quality control method by HPLC which is suitable for Perillae Fructus, Perillae Folium and Perillae Caulis was set up for the first time. The contents of rosmarinic acid in PCa, PFo and PFr samples collected from different herbal markets were compared. This method was simple, fast, easy and accurate. Among the three medicinal parts of *P. frutescens*, PFo had the highest RA content. Results also showed that some macroscopic characteristics were related to the concentration of RA. Samples collected in Hong Kong markets had more consistent and higher concentration of RA than samples collected in mainland China markets. Volatile components extracted from PCa, PFo and PFr were also identified and analyzed for comparison by GC-MS systemically. *β*-caryophyllene, 2-hexanoylfuran, *β*-farnesene, 1,4,7-cycloundecatriene-1,5,9,9-tetramethyl-zzz and 1-cyclohexane-1-carboxaldehyde were common constituents in PCa, PFo and PFr. Additionally, *α*-farnesene and caryophyllene oxide were only found in PFr and PFo. These results offer useful information for the basic comparison between the three medicinal parts of *P. frutescens*.

## Abbreviations

HPLC: High performance liquid chromatography; DAD: Photodiode array detector; GC-MS: Gas chromatography-mass spectrum; PCa: Perillae Caulis; PFo: Perillae Folium; PFr: Perillae Fructus; RA: Rosmarinic acid.

## Competing interests

The authors declare that they have no competing interests.

## Authors’ contributions

HBC initiated and all authors designed the study. The sample extraction was conducted by YLW and JL. The method developments were conducted by JL who drafted the manuscript. All authors contributed to the data analyses and to finalizing the manuscript. All authors have read and approved the final version.

## Supplementary Material

Additional file 1: Table S1 HPLC results by different extraction solvents (sonication for 30 min). Click here for file

Additional file 2: Table S2 HPLC results by different extraction methods. Click here for file

Additional file 3: Table S3 HPLC results by different extraction times. Click here for file

Additional file 4: Figure S1 Resolved mass spectra for main peaks in GC-MS chromatograms of PCa, PFo and PFr. a: 2-hexanoylfuran (8); b: *β*-caryophyllene (10); c: asarone (20). Click here for file
