# Supplementary material for: Determination of the content of rosmarinic acid by HPLC and analytical comparison of volatile constituents by GC-MS in different parts of Perilla frutescens (L.) Britt
Source: Chem Cent J. 2013 Apr 1;7:61. doi: 10.1186/1752-153X-7-61 (PMC3636040; doi:10.1186/1752-153X-7-61)
Supplement: Additional file 1: Table S1 — HPLC results by different extraction solvents (sonication for 30 min). [file 1752-153X-7-61-S1.docx]

**Additional file 1:**

**Table S1.** HPLC results by different extraction solvents (sonication for 30 min).

| **Sample** | **Solvent** | **Weight (g)** | **Peak Area (mAU*s)** | **Peak Area/Weight** |
| --- | --- | --- | --- | --- |
| **PCa-01** | 70% MeOH | 0.5065 | 131.6 | 259.82 |
|  | MeOH | 0.5095 | 63.8 | 125.22 |
|  | 70% EtOH | 0.5044 | 139.6 | 276.76 |
|  | EtOH | 0.5193 | Not detected | - |
| **PFo-01** | 70% MeOH | 0.5076 | 410.2 | 808.12 |
|  | MeOH | 0.5005 | 114.2 | 228.17 |
|  | 70% EtOH | 0.5080 | 396.3 | 760.43 |
|  | EtOH | 0.4981 | Not detected | - |
| **PFr-01** | 70% MeOH | 0.5057 | 1092.7 | 2160.77 |
|  | MeOH | 0.5000 | 148.4 | 296.8 |
|  | 70% EtOH | 0.5006 | 973.8 | 1945.27 |
|  | EtOH | 0.5063 | Not detected | - |
