# Supplementary material for: Determination of the content of rosmarinic acid by HPLC and analytical comparison of volatile constituents by GC-MS in different parts of Perilla frutescens (L.) Britt
Source: Chem Cent J. 2013 Apr 1;7:61. doi: 10.1186/1752-153X-7-61 (PMC3636040; doi:10.1186/1752-153X-7-61)
Supplement: Additional file 2: Table S2 — HPLC results by different extraction methods. [file 1752-153X-7-61-S2.docx]

**Additional file 2:**

**Table S2.** HPLC results by different extraction methods.

| **Sample** | **Method** | **Weight (g)** | **Peak Area (mAU*s)** | **Peak Area /Weight** |
| --- | --- | --- | --- | --- |
| **PCa-01** | Sonication | 0.4986 | 132.4 | 265.54 |
|  | Reflux for 1 h | 0.5050 | 170.1 | 336.83 |
|  | Reflux for 2 h | 0.5046 | 169.5 | 335.91 |
| **PFo-01** | Sonication | 0.5004 | 419.6 | 838.53 |
|  | Reflux for 1 h | 0.5271 | 618.2 | 1172.83 |
|  | Reflux for 2 h | 0.5116 | 606.6 | 1185.69 |
| **PFr-01** | Sonication | 0.4942 | 855.8 | 1731.69 |
|  | Reflux for 1 h | 0.5111 | 1262.6 | 2470.36 |
|  | Reflux for 2 h | 0.5628 | 1425.7 | 2533.23 |
