# Supplementary material for: Determination of the content of rosmarinic acid by HPLC and analytical comparison of volatile constituents by GC-MS in different parts of Perilla frutescens (L.) Britt
Source: Chem Cent J. 2013 Apr 1;7:61. doi: 10.1186/1752-153X-7-61 (PMC3636040; doi:10.1186/1752-153X-7-61)
Supplement: Additional file 3: Table S3 — HPLC results by different extraction times. [file 1752-153X-7-61-S3.docx]

**Additional file 3:**

**Table S3.** HPLC results by different extraction times.

| **Sample** | **Times of Extraction** | **Peak Area (mAU*s)** |
| --- | --- | --- |
| **PCa-01** | 1 | 131.6 |
|  | 2 | 38.1 |
|  | 3 | Not detected |
| **PFo-01** | 1 | 410.2 |
|  | 2 | 69.7 |
|  | 3 | Not detected |
| **PFr-01** | 1 | 1092.7 |
|  | 2 | 175.9 |
|  | 3 | Not detected |
