# Supplementary material for: Determination of the content of rosmarinic acid by HPLC and analytical comparison of volatile constituents by GC-MS in different parts of Perilla frutescens (L.) Britt
Source: Chem Cent J. 2013 Apr 1;7:61. doi: 10.1186/1752-153X-7-61 (PMC3636040; doi:10.1186/1752-153X-7-61)
Supplement: Additional file 4: Figure S1 — Resolved mass spectra for main peaks in GC-MS chromatograms of PCa, PFo and PFr. a: 2-hexanoylfuran (8); b: β-caryophyllene (10); c: asarone (20). [file 1752-153X-7-61-S4.docx]

a

b

c

**Additional file 4:**

**Figure S1.** Resolved mass spectra for main peaks in GC-MS chromatograms of PCa, PFo and PFr. a: 2-hexanoylfuran (8); b: *β*-caryophyllene (10); c: asarone (20).
